# Increased risk of IgE-mediated allergies in patients with celiac disease: a case-control study

**DOI:** 10.3389/fnut.2025.1713019

**Published:** 2026-01-12

**Authors:** Ahemalali Azhati, Shenglong Xue, Tian Shi, Mengmeng Guan, Wenjie Kong, Yan Feng, Xin Ma, Aliya Ayerken, Feng Gao

**Affiliations:** 1 Department of Gastroenterology, People's Hospital of Xinjiang Uygur Autonomous Region, Urumqi, China; 2 Xinjiang Clinical Research Center for Digestive Diseases, Urumqi, China; 3 Xinjiang Clinical and Research Center for Celiac Disease, Urumqi, China; 4 College of Life Science and Technology, Xinjiang University, Urumqi, China; 5 Department of Dermatology and Venereology, People's Hospital of Xinjiang Uygur Autonomous Region, Urumqi, China

**Keywords:** celiac disease, complications, IgE-mediated allergies, Marsh classification, prevalence

## Abstract

**Background:**

Celiac disease (CeD) is an autoimmune small intestinal disorder frequently associated with allergic diseases. However, the prevalence, allergen differences, and clinical characteristics of allergic reactions in adult CeD patients remain unclear.

**Objective:**

The primary aim of this study, conducted primarily in the Chinese population, is to determine the prevalence and clinical characteristics of patients with CeD who have concomitant allergies.

**Methods:**

A total of 92 CeD patients diagnosed between June 2018 and June 2025 and 184 healthy subjects matched for age and gender were enrolled. Allergen profiles were evaluated in both CeD patients and healthy controls to compare the prevalence of allergies and differences in major allergens between the two groups. A retrospective analysis of clinical data and medical records from 92 patients with CeD was performed to further explore the clinical characteristics, prevalence of allergic comorbidities, and endoscopic Marsh classification features in patients with CeD who had and did not have allergies.

**Results:**

The prevalence of IgE-mediated allergies in CeD patients was 58.7% (54/92), which was significantly higher than that in healthy controls (24.5%, 45/184) (*P* < 0.05). Allergen testing showed that the dominant allergens in CeD patients included milk, beef, wheat flour, and dog epithelium. Additionally, among the CeD patients, the prevalence of allergic rhinitis was 2.17% (2/92), allergic asthma was 1.09% (1/92), and atopic dermatitis was 5.43% (5/92). Compared with CeD patients without comorbid allergies, those with allergies had a significantly lower body mass index (BMI) (22.89 ± 5.21 vs. 20.92 ± 4.21 kg/m^2^, *P* < 0.05) and a substantially higher incidence of diarrhea (15.79% vs. 38.89%, *P* < 0.05). No significant differences were found between the two groups in terms of gender distribution, tissue transglutaminase IgA (tTg-IgA) levels, or Marsh classification.

**Conclusion:**

The results of this study demonstrate that the prevalence of IgE-mediated allergies in patients with CeD is significantly higher than that in the healthy population. Importantly, comorbid allergies may exacerbate intestinal malabsorption and worsen digestive symptoms (specifically diarrhea) in CeD patients. Based on these findings, allergen screening is strongly recommended for CeD patients presenting with severe malabsorption or refractory diarrhea.

## 1 Introduction

Celiac disease (CeD), also known as gluten-sensitive enteropathy, is an autoimmune disorder of the small intestine that is primarily triggered by the ingestion of gluten proteins in genetically susceptible individuals (1). CeD affects approximately 0.5% to 2.0% of the global population, with a serologically diagnosed prevalence of about 1.4% (1, 2). It can develop at any stage of life. The pathogenesis of CeD is driven by a complex interplay between the innate and adaptive arms of the immune system (3, 4). On the innate immunity front, undigested gluten peptides (such as the 33-mer and 13-mer gliadin peptides) increase intestinal permeability through activation of the zonulin pathway and induce endoplasmic reticulum stress and oxidative stress. These immune reactions stimulate intestinal epithelial cells to release pro-inflammatory cytokines, including IL-15 and IL-8, which in turn activate intraepithelial lymphocytes, leading to enterocyte damage and the establishment of an early inflammatory microenvironment (3, 4). For adaptive immunity, HLA-DQ2/DQ8 molecules are recognized by antigen-presenting cells (APCs), thereby triggering specific CD4+ T cell-mediated immune responses and initiating Th1-type immune reactions (5–8). This process promotes the release of inflammatory cytokines such as IFN-γ, IL-12, and TNF-α, destroying intestinal villi (5–8). The clinical manifestations of CeD are heterogeneous, with the primary symptoms including abdominal pain, chronic diarrhea, bloating, and weight loss. Currently, a gluten-free diet (GFD) remains the sole effective therapeutic approach for CeD. Nevertheless, some patients continue to experience symptoms such as abdominal pain and bloating despite long-term compliance with a GFD (9). The 2017 Global Guidelines for CeD issued by the World Gastroenterology Organization (WGO) (10) have identified IgE-mediated allergy as one of the causes of persistent gastrointestinal symptoms in CeD patients after initiating a GFD.

In contrast to CeD, IgE-mediated allergic reactions represent a Th2-type immune response, which is an abnormal immunological reaction to exogenous proteins. Related disorders include allergic rhinitis (AR), allergic asthma (AA), atopic dermatitis (AD), and food allergy (FA), among others (11). The pathogenesis involves two main phases: the sensitization phase and the effector phase (12). During the sensitization phase, allergens are captured by APCs and presented via MHC class II molecules, prompting the differentiation of naive Th0 cells into Th2 cells. These activated Th2 cells subsequently secrete IL-4 and IL-13, which drive B cells to produce allergen-specific IgE antibodies. In the effector phase, re-exposure to the same allergen triggers the cross-linking of IgE antibodies bound to high-affinity receptors on mast cells and basophils, leading to the release of bioactive mediators and the onset of allergic symptoms. Gastrointestinal manifestations of IgE-mediated allergies primarily include abdominal pain, diarrhea, and abdominal discomfort (12–15).

Although CeD and IgE-mediated allergic reactions are fundamentally distinct in their pathogenesis, a growing body of research has recently begun to explore the potential relationship between the two. A recently published systematic review has examined this association, and multiple studies indicate that the prevalence of IgE-mediated allergic reactions is significantly higher in CeD patients compared to the general population (16–18). Specifically, Kårhus LL et al. observed that patients with positive CeD antibodies or biopsy-confirmed CeD had higher rates of IgE-mediated allergies to specific food and inhalant allergens than healthy subjects (16). An earlier single-center study focusing on pediatric CeD patients reported that 20.3% of CeD children had IgE-mediated allergic reactions; among these allergic children, 58.3% were sensitized to food allergens and 66.7% to airborne allergens. Simultaneously, the same study indicated that gastrointestinal symptoms in 41.7% of pediatric CeD patients were associated with the ingestion of allergenic foods, which implies a potential association between CeD and IgE-mediated allergic reactions (17). However, current research on the correlation between CeD and IgE-mediated allergic reactions is still limited, with a primary focus on pediatric CeD populations (17, 19, 20). The prevalence, allergen spectrum, and clinical characteristics of IgE-mediated allergic reactions in patients with CeD remain incompletely characterized. Moreover, no studies to date have specifically investigated IgE-mediated allergies in Chinese CeD patients.

In this study, we systematically assessed the prevalence of IgE-mediated allergic reactions and allergen differences between Chinese adult CeD patients and healthy controls. We further analyzed whether CeD patients with concurrent allergic reactions exhibited differences in clinical symptoms, tTg-IgA concentrations, and severity of intestinal mucosal damage. This study aims to provide evidence for individualized management strategies in CeD patients and offer clinical recommendations for CeD patients with persistent gastrointestinal symptoms despite GFD.

## 2 Methods

### 2.1 Research object

**Figure 1:**
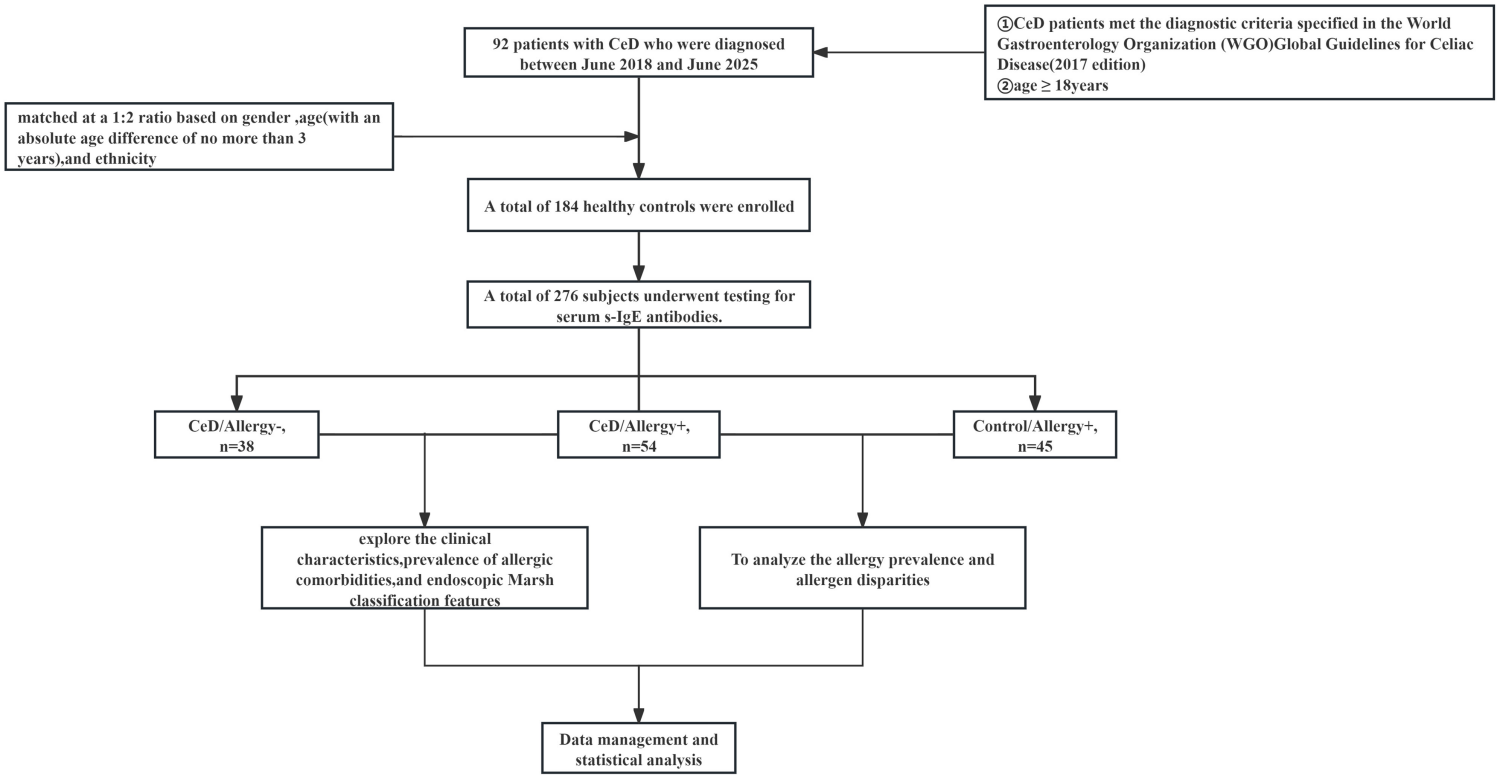
Overall study design.

The overall design and experimental workflow of this study are illustrated in Figure 1. This study is a case-control investigation in which the sample size was not determined by a priori power calculation. The study consecutively included 92 adult patients with CeD who were diagnosed at the Department of Gastroenterology, People's Hospital of Xinjiang Uygur Autonomous Region, between June 2018 and June 2025. These patients were matched at a 1:2 ratio with 184 healthy controls based on sex, age (with a maximum absolute difference of 3 years), and ethnicity. All CeD patients fulfilled the diagnostic criteria specified in the World Gastroenterology Organization (WGO) Global Guidelines for Celiac Disease (2017 edition) (10). The inclusion criteria for CeD patients were: positive serum anti-tissue transglutaminase immunoglobulin A (tTg-IgA) (with a tTg-IgA level ≥ 20 U/ml), completion of gastroscopy with histopathological examination confirming Marsh stage ≥ II, and age ≥ 18 years. Blood samples from all patients with CeD were archived from previous research projects.

The healthy control subjects were recruited from the Physical Examination Center of the People's Hospital of Xinjiang Uygur Autonomous Region. All participants were in good general health, without current health complaints or history of acute/chronic diseases, and had provided blood samples. Through physician interviews or telephone contacts, they were informed that their blood samples would be used for allergen screening and celiac disease research. Testing was performed only after obtaining signed informed consent. Ultimately, only individuals with negative tissue transglutaminase (tTG) antibody results (using a cutoff value of tTG-IgA ≤ 1.9 U/mL) were included in the healthy control group. All subjects (including CeD patients and healthy controls) met the following exclusion criteria: (1) Incomplete clinical baseline data, such as missing medical history records or unavailable endoscopic findings; (2) Comorbid gastrointestinal disorders (e.g., inflammatory bowel disease [IBD], irritable bowel syndrome [IBS]), malignant tumors, or severe infectious diseases; (3) Current use of medications that may affect immune function or intestinal mucosal integrity (e.g., immunosuppressive agents, long-term high-dose antibiotics); (4) Use of topical corticosteroids within the previous 2 weeks, or oral antihistamines or corticosteroids within the previous 2 months.

This study was reviewed and approved by the Ethics Committee of Xinjiang Uygur Autonomous Region People's Hospital, with the ethics approval number KY202412087. All participants voluntarily participated in the study and signed written informed consent forms before enrollment.

### 2.2 Evaluation criteria of marsh classification in CeD patients

For the pathological diagnosis of CeD, small intestinal mucosal lesions were graded in accordance with the Marsh-Oberhuber histopathological classification system (21), with specific grades defined as follows: Grade 0 (normal): Characterized by a normal small intestinal mucosal structure; Grade I (intraepithelial lymphocytosis): Defined by an increased number of intraepithelial lymphocytes (typically >25 lymphocytes per 100 enterocytes) without other mucosal abnormalities; Grade II (intraepithelial lymphocytosis with crypt hyperplasia): Presented with increased intraepithelial lymphocytes accompanied by crypt hyperplasia (elevated crypt depth and cellularity); Grade IIIa (partial villous atrophy): Exhibited partial loss of villous height; Grade IIIb (near-total villous atrophy): Showed nearly complete villous loss; Grade IIIc (total villous atrophy): Demonstrated complete villous atrophy. A confirmed CeD diagnosis required the pathological grading of small intestinal biopsy specimens to be Marsh ≥ Grade II.

### 2.3 Assay of IgE antibodies and diagnostic criteria for IgE-mediated allergic diseases

This study primarily relies on serum-specific IgE (s-IgE) testing and clinical history collection to evaluate IgE-mediated allergic status. Although the skin prick test (SPT) is a common *in vivo* method for diagnosing IgE-mediated allergies, it was not uniformly included as a diagnostic procedure in this study due to its retrospective design, which depended on archived serum samples and medical records. Additionally, SPT was not routinely performed for patients during their clinical visits. s-IgE Antibody Detection: All subjects underwent an 8–10-h overnight fast. The following morning, 3 mL of venous blood was collected from each subject. Blood samples were stored at 2–8 °C and analyzed within 48 h of collection. If samples required storage beyond 48 h, they were frozen at -−20 °C (or below); before testing, frozen samples were thawed at room temperature (20–25 °C) and gently inverted repeatedly to ensure thorough mixing. All serum samples were inspected to exclude potential interfering factors such as hemolysis and lipemia that could compromise assay accuracy. S-IgE levels were quantitatively measured using a commercial kit (Product No.: MB00145) manufactured by Jiangsu Haobo Biological Medicine Co., Ltd. (Suzhou, China). According to the manufacturer's specifications, the assay has a lower detection limit of ≤ 0.35 IU/mL, demonstrates excellent linearity within the range of 0–100 IU/mL (r > 0.99), and exhibits a precision profile with a coefficient of variation (CV) ≤ 15%. All testing procedures were performed in strict accordance with the standardized operating protocol, with internal quality controls included in each run. An sIgE level ≥ 0.35 IU/mL was defined as a positive result. Furthermore, the panel of 20 allergens tested in this study was predetermined by the commercial assay kit (MB00145) used. The selection of these allergens is grounded in robust regional epidemiological evidence and is designed to cover the most prevalent and clinically relevant inhalant and food allergens in the Chinese population (22). The allergens included in the kit were categorized into two groups: (1) Food allergens: peanut, soybean, milk, crab, shrimp, egg, beef, cod, wheat flour, and lamb; (2) Inhalable allergens: Dermatophagoides pteronyssinus, Dermatophagoides farinae, dog epithelium, cat epithelium, cockroach, Aspergillus spp., willow, common ragweed pollen, mugwort pollen, and house dust. In this analysis, to maintain consistency with the test report, Dermatophagoides pteronyssinus and Dermatophagoides farinae are counted as two separate allergens in the allergy count.

Serological Diagnostic Criteria for s-IgE Positivity: A serum s-IgE antibody concentration ≥0.35 IU/mL was defined as a positive result for the corresponding allergen.

Diagnostic Criteria for Allergic Diseases: Detailed clinical data were collected for all patients with CeD. A history of allergic asthma (AA) or allergic rhinitis (AR) was confirmed based on patients' self-reported symptoms and medical records. For patients with CeD who presented with skin allergy symptoms and consulted a dermatologist, skin biopsies were performed when clinically indicated and active skin lesions were present, serving to assist with diagnosis or differential diagnosis. To ensure diagnostic accuracy, CeD patients whose biopsy results confirmed bullous dermatitis or contact dermatitis (attributed to prolonged irritant exposure) were excluded from the atopic dermatitis (AD) diagnostic cohort.

### 2.4 Statistical analysis

Data analysis in this study was performed using SPSS 26.0 software. For continuous data, independent samples t-tests were used to assess differences. For data not following a normal distribution, median (interquartile range) was used for description, and nonparametric Mann-Whitney U tests were employed for intergroup comparisons. For unordered count data, frequencies (percentages %) were used for description. Count data were analyzed using chi-square analysis (between-group comparisons, including the continuity correction method and Fisher's exact probability test). For ordered count data, frequencies (percentages %) were used for description, and differences were analyzed using the nonparametric Mann-Whitney U test (between-group comparisons). *P* < 0.05 was considered statistically significant. Additionally, to evaluate the statistical power of the present study, a *post hoc* analysis was conducted using G^*^Power 3.1. The analysis revealed a power of >99.9% (α = 0.05) for detecting the observed effect.

## 3 Results

### 3.1 Baseline data of CeD patients, healthy controls

This study enrolled 92 adult patients with CeD and 184 healthy control subjects (Con), with detailed baseline characteristics summarized in Table 1. Regarding age, the CeD group had a mean ± standard deviation (SD) age of 49.23 ± 13.18 years, while the Con group had a mean ± SD age of 49.10 ± 12.98 years—indicating comparable age distributions between the two groups. In terms of gender, both groups had identical gender proportions: males accounted for 28.26% (26/92 in the CeD group, 52/184 in the Con group), and females accounted for 71.74% (66/92 in the CeD group, 132/184 in the Con group), resulting in a male-to-female ratio of approximately 1:2.5 in both cohorts. For the CeD group, body mass index (BMI) values ranged from 13.30 to 40.90 kg/m^2^, with a mean BMI of 21.73 kg/m^2^.

**Table 1:** Baseline characteristics of the CeD group and control group, and differences in allergy prevalence between the two groups.

**Parameters**	**CeD (*n* = 92)**	**Control (*n* = 184)**	**Statistical value**	***P*-value**
**Age**				
	49.23 ± 13.18	49.10 ± 12.98	-	-
**Gender**			-	-
Male	26 (28.26%)	52 (28.26%)	-	-
Female	66 (71.74%)	132 (71.74%)	-	-
CeD/Allergy^+^	54 (58.70%)	45 (24.46%)	*χ^2^ =* 31.257	< 0.001
CeD/Allergy^−^	38 (41.30%)	139 (75.54%)		
Food allergens	44 (47.83%)	18 (9.78%)	*χ^2^ =* 50.965	< 0.001
Inhalant allergens	25 (27.17%)	31 (16.85%)	*χ^2^ =* 4.044	0.044
More than one allergen	28 (30.43%)	16 (8.70%)	*χ^2^ =* 21.63	< 0.001

### 3.2 Analysis of IgE-mediated allergy prevalence and allergy status in the CeD patients and healthy controls

Differences in the prevalence of IgE-mediated allergies between the two groups are summarized in Table 1. In the CeD group, 58.7% of patients (54/92) were diagnosed with IgE-mediated allergies, whereas the incidence of sensitization in the Con group was only half that of the CeD group, at 24.5% (45/184); this between-group difference was statistically significant (*P* < 0.05). It is noteworthy that most CeD patients (54/92) were allergic to at least one allergen. Differences in the allergen spectrum (i.e., distribution of specific allergens) between the two groups are detailed in Table 2. For food allergens, the highest allergy rates in the CeD group were for beef and wheat flour, both at 25.0% (23/92), followed by milk (13.1%, 12/92) and eggs (8.7%, 8/92). In contrast, the Con group had a very low food allergen sensitization rate, with eggs being the most common food allergen (only ~2.72%, 5/184). Regarding inhalant allergens, the most prevalent in the celiac disease group was dog epithelium, with an allergy rate of 10.87% (10/92). In the Con group, the most common inhalant allergen was mugwort, with a sensitivity rate of 5.43% (10/184).

**Table 2:** Differences in allergens between the CeD group and the control group.

**Allergens**		**CeD (*n* = 92)**	**Control (*n* = 184)**	**Statistical value**	***P*-value**
**Food allergens**					
	Peanut	0 (0.00%)	1 (0.54%)	-	1.000
	Soybean	0 (0.00%)	3 (1.63%)	*χ^2^ =* 0.379	0.538
	Milk	12 (13.14%)	1 (0.54%)	*χ^2^ =* 18.658	< 0.001
	Crab	2 (2.17%)	4 (2.17%)	*χ^2^ =* 0.000	1.000
	Shrimp	1 (1.09%)	0 (0.00%)	-	0.333
	Eggs	8 (8.70%)	5 (2.72%)	*χ^2^ =* 3.643	0.056
	Beef	23 (25.00%)	1 (0.54%)	*χ^2^ =* 46.205	< 0.001
	Cod	0 (0.00%)	0 (0.00%)	-	-
	Wheat flour	23 (25.00%)	1 (0.54%)	*χ^2^ =* 46.205	< 0.001
	Lamb	0 (0.00%)	4 (2.17%)	*χ^2^ =* 0.793	0.373
**Inhalant allergens**					
	Dermatophagoides pteronyssinus	7 (7.61%)	9 (4.89%)	*χ^2^ =* 0.829	0.362
	Dermatophagoides farinae	6 (6.52%)	9 (4.89%)	*χ^2^ =* 0.317	0.573
	Dog epithelium	10 (10.87%)	5 (2.27%)	*χ^2^ =* 7.931	0.005
	Cat epithelium	0 (0.00%)	6 (3.26%)	*χ^2^ =* 1.725	0.189
	House dust	0 (0.00%)	3 (1.63%)	*χ^2^ =* 0.379	0.538
	Cockroach	4 (4.35%)	3 (1.63%)	*χ^2^ =* 0.898	0.343
	Alternaria	0 (0.00%)	0 (0.00%)	-	-
	Willow	2 (2.17%)	3 (1.63%)	*χ^2^ =* 0.000	1.000
	Common ragweed	2 (2.17%)	2 (1.09%)	*χ^2^ =* 0.032	0.859
	Mugwort	4 (4.35%)	10 (5.43%)	*χ^2^ =* 0.009	0.923

### 3.3 Distribution of allergic diseases in CeD

**Figure 2:**
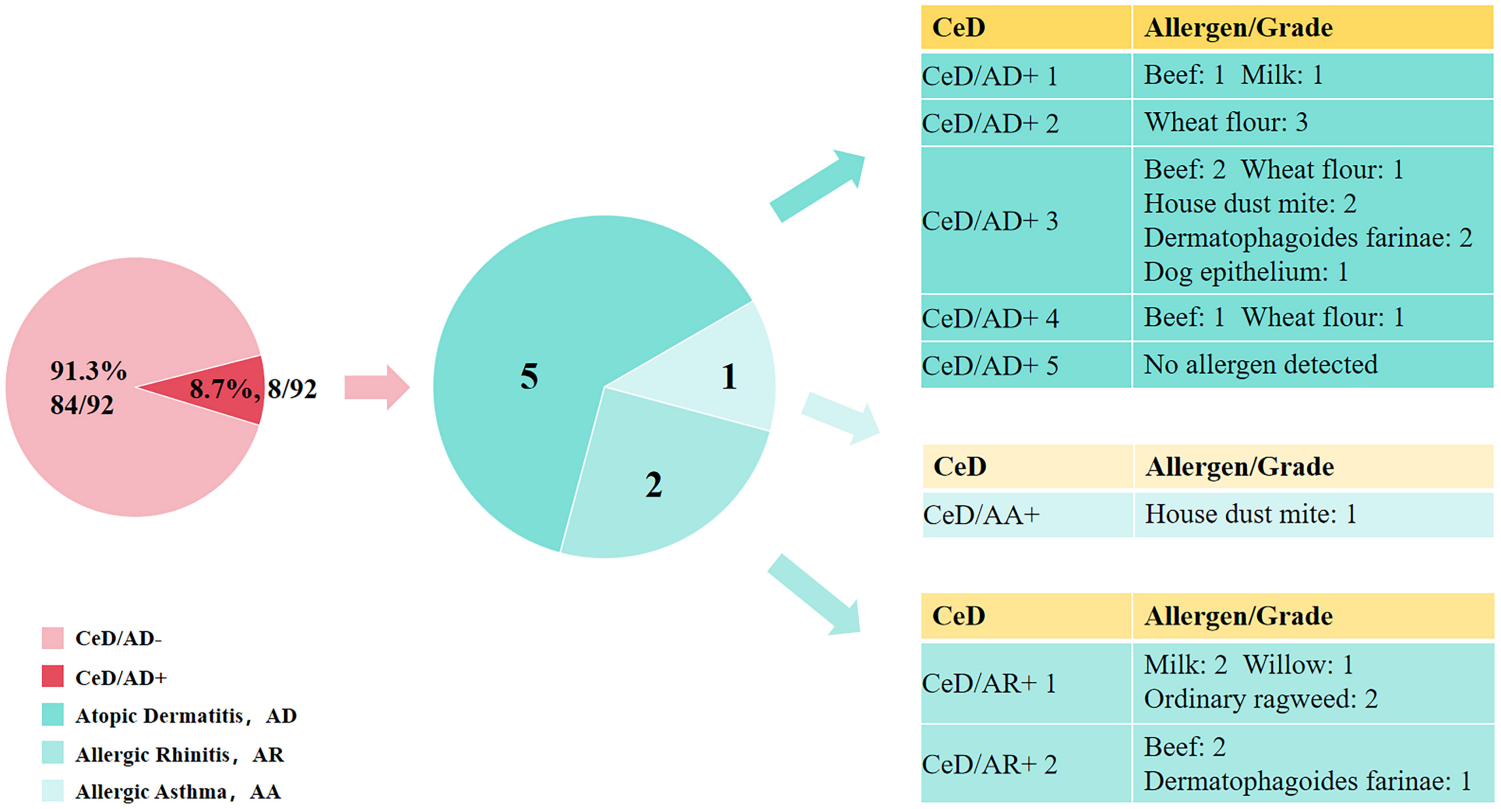
Distribution of allergic diseases in CeD.

Comorbid IgE-mediated allergic diseases in adult CeD patients were statistically analyzed based on their medical histories (including past allergic disease diagnoses) and clinical manifestations recorded during hospitalization. The analyzed diseases included allergic asthma (AA), allergic rhinitis (AR), and atopic dermatitis (AD). Results showed that the prevalence of these comorbid allergic diseases among adult CeD patients was 1.09% (1/92) for AA, 2.17% (2/92) for AR, and 5.43% (5/92) for AD; detailed data are presented in Figure 2. Among the 8 CeD patients with comorbid allergic diseases, 7 tested positive for at least one specific allergen, and among these 7 allergen-positive patients, 5 were allergic to multiple allergens. The distribution of specific allergens in the 7 positive patients was as follows: For inhalant allergens, 2 patients were allergic to Dermatophagoides pteronyssinus and Dermatophagoides farinae, respectively; 1 patient was allergic to dog epithelium, 1 to willow pollen, and 1 to common ragweed pollen. Only 1 patient with comorbid allergic disease tested negative for all specific allergens included in the detection panel; detailed data are presented in Figure 2.

### 3.4 Analysis of clinical characteristics of CeD with and without comorbid allergy

To explore the association between IgE-mediated allergies and clinical characteristics of CeD, the CeD group was further stratified into two subgroups: the CeD-allergy subgroup and the CeD-non-allergy subgroup. Five key clinical indicators were compared between the two subgroups: age, body mass index (BMI), serum tTg-IgA concentration, endoscopic Marsh classification, and clinical symptoms; detailed results are summarized in Table 3. No statistically significant differences were detected between the two subgroups in terms of age, distribution of serum tTg-IgA concentrations, and endoscopic Marsh classification (all *P* > 0.05). In contrast, a significant between-subgroup difference was observed in BMI (*P*=0.048): the CeD-allergy subgroup had a lower mean BMI (20.92 ± 4.21 kg/m^2^) compared to the CeD-non-allergy subgroup (22.89 ± 5.21 kg/m^2^), and this difference remained significant when re-verified (*P* < 0.05). For clinical symptoms, the prevalence of diarrhea differed significantly between the two subgroups (38.9% in the CeD-allergy subgroup vs. 15.8% in the CeD-non-allergy subgroup, *P* = 0.017). No significant between-subgroup differences were found in other common CeD-related symptoms, including abdominal pain, bloating, fatigue, loss of appetite, and weight loss (*P* > 0.05).

**Table 3:** Demographic data, clinical symptoms, and marsh classification between CeD/allergy+ and CeD/allergy- groups.

**Parameters**	**CeD/Allergy+(*n* = 54)**	**CeD/Allergy-(*n* = 38)**	**Statistical value**	***P*-value**
Age, mean ± SD	47.59 ± 11.03	51.55 ± 15.60	t =1.346	0.183
BMI, mean ± SD	20.92 ± 4.21	22.89 ± 5.21	t =2.005	0.048
Ttg, M(Q1, Q3)	1,079.30 (259.82, 2,717.40)	1,425.10 (248.30, 2,550.00)	Z =-0.368	0.713
**Clinical symptoms**				
Abdominal pain, *n* (%)	29 (53.70%)	15 (39.47%)	χ^2^ = 1.810	0.179
Diarrhea, *n* (%)	21 (38.89%)	6 (15.79%)	χ^2^ = 5.740	0.017
Abdominal distension, *n* (%)	20 (37.04%)	9 (23.68%)	χ^2^ = 1.842	0.175
Fatigue, *n* (%)	17 (31.48%)	8 (21.05%)	χ^2^ = 1.226	0.268
Anorexia, *n* (%)	8 (21.05%)	12 (22.22%)	χ^2^ = 0.018	0.893
Weight loss, *n* (%)	24 (44.44%)	15 (39.47%)	χ^2^ = 0.226	0.635
**Marsh classification**, ***n*** **(%)**				
Marsh II	4 (7.41%)	8 (21.05%)	Z = −0.052	0.958
Marsh IIIa	11 (20.37%)	5 (13.16%)		
Marsh IIIb	29 (53.70%)	15 (39.47%)		
Marsh IIIc	10 (18.52%)	10 (26.32%)		

## 4 Discussion

CeD is a T-cell-mediated autoimmune enteropathy that occurs in susceptible individuals carrying human leukocyte antigen (HLA)-DQ2 and/or HLA-DQ8 after gluten intake. Its pathogenesis is well recognized as multifactorial, involving the interplay of genetic predisposition, dysregulated mucosal immune responses, and environmental triggers (7, 23). By contrast, IgE-mediated allergic reactions are type I hypersensitivity responses characterized by excessive immune activation against exogenous proteins (allergens); in this process, the immune system erroneously classifies normally tolerable substances as pathogenic, leading to IgE production, mast cell degranulation, and the release of pro-inflammatory mediators (e.g., histamine) (24). Although the pathogenesis of CeD and IgE-mediated allergic reactions is different, existing evidence indicates that IgE-mediated allergic reactions are highly prevalent among CeD patients (16, 17, 20, 25). Moreover, studies have shown that the two share similar genetic backgrounds, with HLA-DQ2 and/or DQ8 being the main common genetic factors for CeD and IgE-mediated allergic reactions (26, 27). However, current research mainly focuses on children, and there are relatively few studies on the association between adult CeD and IgE-mediated allergies, especially regarding the prevalence of allergies and their clinical characteristics in adult CeD patients. Therefore, this study systematically evaluated the prevalence of IgE-mediated allergies in adult CeD patients in the Chinese population and further analyzed the clinical characteristics of adult CeD patients with concurrent allergies. This research, to some extent, fills the gap in clinical data for Chinese CeD patients with concurrent allergies.

This study retrospectively analyzed the prevalence of allergic diseases and clinical characteristics of adult CeD patients by measuring serum s-IgE antibodies in 92 CeD adult patients and 184 healthy individuals. In this study, we found that the overall prevalence of allergy in adult CeD patients was 58.7% (54/92), while the prevalence of sensitization in the healthy control group was 24.5% (45/184) (*P* < 0.05). The prevalence of IgE-mediated allergic reactions in adult CeD patients was significantly higher than that in the general population. More than half of the CeD patients had one or more allergies. We hypothesize that this high comorbidity rate may involve the synergistic effects of multiple factors, such as the intestinal barrier, immune regulation, intestinal microbiota, and genetic factors. Firstly, increased intestinal permeability is considered an important inducing factor for the interaction between the two diseases. Genetically susceptible individuals (such as those carrying HLA-DQ2/DQ8 genes) often show decreased expression of intestinal epithelial tight junction proteins (such as occludin, claudin-3/4, and ZO-1), leading to increased intestinal permeability (28, 29). This “leaky gut” phenomenon not only accelerates the trans-epithelial translocation of gluten-derived peptides but also enhances the penetration of food allergens across the intestinal mucosal barrier—allowing these allergens to reach the underlying lamina propria and interact with immune cells, thereby initiating IgE-mediated allergic responses (25). The “leaky gut” phenomenon not only promotes the transepithelial translocation of gluten protein peptides but also enables food allergens to cross the mucosal barrier more easily, ultimately triggering allergic responses (25). Secondly, both CeD and IgE-mediated allergy are associated with the body's immune response. Research has proposed that immune system dysregulation may already exist in CeD patients, making them more prone to developing allergies to different food allergens (30). In addition, CeD and IgE-mediated allergy share a common genetic basis. A prospective observational study demonstrated that children carrying the HLA-DQ2/8 genes had heightened s-IgE sensitivity by the age of 3 and a higher risk of allergies to common allergens than the general pediatric population (26). Consequently, the high incidence of allergic diseases in CeD patients is not the outcome of a single factor but rather the consequence of the interaction of multiple factors.

CeD is dominated by a Th1-type immune response, while IgE-mediated allergic reactions are primarily driven by a Th2-type immune response. Early research exploring the correlation between CeD and IgE-mediated allergic diseases has focused predominantly on CeD comorbidity profiles, with specific attention to associations with allergic asthma (AA), allergic rhinitis (AR), and atopic dermatitis (AD) (31–34). In the present study, we evaluated the prevalence of these conditions in adult CeD patients and performed a descriptive comparison with published data from the general population (34, 35). Our results showed that among adult CeD patients, the prevalence of AA was 1.09% (1/92) and that of AR was 2.17% (2/92)—both figures falling at or below the ranges reported for the general adult population. Previous studies have indicated that the prevalence of AA in general adults is approximately 5%−10%, while that of AR ranges from about 10% to 40% (34, 35). In contrast, the prevalence of AD in our CeD group was 5.43% (5/92), which appears numerically higher compared to the 1%−3% reported for AD in general adults (34, 35). Multiple earlier studies have also found no significant increase in the incidence of IgE-mediated allergic conditions such as AA and AR among CeD patients, suggesting no obvious association (31, 33).In contrast, some studies have identified a certain correlation between CeD and AA: a cohort study involving 28,281 CeD patients and 140,295 non-CeD patients demonstrated that the risk ratio of AA in CeD patients relative to non-CeD patients was 1.61 (32). Notably, a cross-sectional observational study demonstrated a significant association between AD and CeD (34), a finding that appears consistent with the elevated AD prevalence trend observed in our study.

Furthermore, we conducted a more in-depth analysis of the allergen profile in CeD patients. The results showed that the common food allergens in the CeD group were wheat flour (25%, 23/92) and beef (25%, 23/92), followed by milk (13%, 12/92) and eggs (8.7%, 8/92). In contrast, approximately 2.72% (5/184) of the healthy control group were sensitive to eggs. The most common inhalant allergen in CeD patients was dog epithelium (10.87%, 10/92), whereas in the healthy control group, it was mugwort (5.83%, 10/184). The allergies to wheat flour, milk, and eggs in CeD patients were consistent with the findings of previous studies on allergens in CeD patients (16, 36). Hasna Ait et al. reported that 48% (27/57) of CeD children had IgE-mediated allergies; the primary allergen was chicken (51.9%, 14/27), followed by almonds (40.7%, 11/27), sesame (40.7%, 11/27), potatoes (33.3%, 9/27), and apples (18.5%, 5/27) (20). A study conducted in Poland also found that 20.3% (12/59) of CeD children were diagnosed with IgE-mediated allergies: among these allergy-complicated children, approximately 58.3% (7/12) were allergic to food, and 66.7% (8/12) were allergic to aeroallergens. Specifically, the food allergens mainly included peanuts (41.7%, 5/12), milk (25.0%, 3/12), and egg white (16.7%, 2/12), while the inhalant allergens were primarily mites (50%, 6/12), pollen (41.7%, 5/12), and birch (33.3%, 4/12) (17). Additionally, a study in Iran revealed that 68% (17/25) of CeD patients were allergic to shellfish, and 16% (4/25) were allergic to sesame (30). Variations in geographical location and dietary practices may lead to differences in the prevalence of comorbid allergies in CeD patients. Nevertheless, a synthesis of the aforementioned findings demonstrates that CeD patients have a significantly higher likelihood of developing comorbid allergies compared to healthy individuals, with environmental and dietary exposures potentially serving as the key drivers of IgE-mediated allergic response patterns in CeD patients.

Wheat is the most prominent allergen in CeD patients with comorbid allergies. Wheat contains both protein and non-protein components; ingestion of wheat may trigger diseases such as IgE/non-IgE-mediated allergy, autoimmune response, and gluten intolerance with unknown mechanisms. A common feature of these diseases is the induction of inflammation targeting wheat proteins (37). CeD and wheat allergy (WA) are two distinctly different diseases with independent pathological mechanisms; however, studies have shown that CeD and WA can coexist (38–40). One case report documented two CeD patients on a GFD who developed allergic reactions following accidental wheat exposure. Both patients underwent skin prick tests and s-IgE detection, leading to a diagnosis of WA (41). Another case report identified a CeD patient who, after adhering to a GFD for a period, experienced a life-threatening allergic reaction following accidental ingestion of wheat-containing bread and was diagnosed with WA (40). In this study, the prevalence of WA was significantly higher in CeD patients than in healthy individuals (25% vs. 0.54%). We hypothesize that the emergence of WA after starting a GFD might result from decreased wheat tolerance in CeD patients post-GFD. Although GFD is a well-established effective treatment for CeD, long-term absence of wheat proteins due to GFD weakens the immune system's tolerance to wheat antigens; conversely, it enhances Th2-type immune responses and promotes IgE sensitization (41, 42). Furthermore, we consider that another major factor underlying the coexistence of CeD and WA could be microbial transglutaminase (mTg)—a substance widely utilized in the food industry. mTg is capable of modifying proteins like gluten: through deamidation, it produces gluten hydrolysates with increased intestinal wall permeability, and through cross-linking, it forms novel epitopes, both of which contribute to the development and progression of CeD (43, 44). Notably, the gluten hydrolysates generated by mTg may also trigger cutaneous allergic reactions. For instance, in Japan, soap containing mTg-processed gluten hydrolysates led to percutaneous sensitization in 22 patients, resulting in wheat-dependent exercise-induced anaphylaxis (WDEIA) accompanied by elevated s-IgE levels against the novel epitopes (45). Consequently, some research has identified mTg as a common link between WA and CeD (46).

Few previous studies have focused on CeD complicated with beef allergy, and even fewer have reported the prevalence of beef allergy in CeD patients. However, in the present study, we found that the prevalence of CeD combined with beef allergy was as high as 25% (23/92). CeD patients with this comorbid allergy showed typical clinical symptoms, with gastrointestinal symptoms most commonly including abdominal pain (11/23), diarrhea (9/23), and bloating (8/23), along with fatigue (10/23) and weight loss (11/23). Additionally, allergy-related manifestations such as AR(1 case) and AD(3/23) were observed. Further analysis of the key reasons suggests that this may be related to the specific geographical environment and dietary habits. The CeD patients enrolled in this study were mainly from the Xinjiang Uygur Autonomous Region of China, where the local dietary pattern is dominated by beef, mutton, and high-gluten foods (47). A study based on the European population indicated that myosin light chain 1 (MYL1) and myosin light chain 3 (MYL3) in cooked beef extracts have been identified as the allergen Bos d13 (48). MYL1 and MYL3 possess thermal stability and can cross intestinal epithelial cells in a folded state— a process that does not rely on disrupting the integrity of tight junctions— thereby effectively triggering the sensitization process (48). This may be one of the reasons for the high prevalence of beef allergy in CeD patients. However, the high prevalence of beef allergy observed in celiac patients in northwest China is preliminarily thought to be closely related to the local diet, which is mainly based on beef and mutton. Nevertheless, the specific allergenic protein components and their association with clinical characteristics still need to be further verified in Asian populations. Secondly, previous allergy studies have mostly focused on gluten-related diseases or common allergens in children (e.g., eggs, milk, peanuts), with insufficient systematic screening for red meat allergens such as beef. This may have led to the underestimation of beef's role as an allergen in CeD.

Beyond WA and beef allergy, this study also demonstrated that CeD patients had a significantly higher prevalence of milk protein allergy (CMPA) and dog epithelium allergy compared to healthy individuals (*P* < 0.05). While egg allergy did not reach statistical significance, approximately 8.7% (8/92) of adult CeD patients in this research were found to have comorbid egg allergy. Milk and eggs are typical childhood allergens. The detection of s-IgE positivity in the adult celiac disease cohort in this study may reflect two situations: one possibility is that food allergies persist into adulthood in a small number of patients; another possibility is that it stems from immune memory from childhood allergies, or from immune responses triggered by continuous exposure to food proteins when the intestinal mucosal barrier function is impaired. Considering the 13.1% milk allergy rate and 8.7% egg allergy rate observed in this study, we believe that the allergen profile of adult CeD patients is not simply a direct extension of the allergy patterns seen in children, but may represent a unique immune characteristic related to the pathophysiological changes of the disease itself. However, this situation still needs to be validated through more detailed experimental designs, such as combining clinical diagnostic methods like skin prick tests (SPT) and food challenge (OFC) tests, as well as further clarifying *in vitro* immune mechanism studies. Prior studies have reported that roughly 50% of patients adhering to a GFD display marked mucosal inflammatory reactions to milk proteins. This is characterized by neutrophil activation after rectal challenge, which manifests as increased myeloperoxidase (MPO) release and enhanced nitric oxide (NO) synthesis, with the degree of elevation comparable to that induced by gluten challenge (49). Casein, a milk component, serves as the key sensitizing agent in CMPA. Specifically, the target fractions α-casein (39 kDa) and β-casein (28 kDa) can be identified by serum IgA from CeD patients. Importantly, these casein fractions exhibit peptide homology with gluten proteins (e.g., β-casein contains peptide sequences similar to those of gluten), implying potential cross-reactivity between CeD and CMPA, as both conditions may involve identical pathogenic peptides (50). Furthermore, both casein and gluten are glutamine-rich and can form complexes through tTG, which in turn activates autotoxic immune responses—highlighting shared immunological characteristics between the two (51).

The clinical symptoms of CeD are primarily caused by gluten-induced intestinal villous atrophy, which leads to various gastrointestinal symptoms in CeD patients, including diarrhea, abdominal pain, bloating, and malabsorption. In the present study, we further investigated the clinical manifestations and degree of intestinal mucosal injury (assessed via Marsh classification) between CeD patients with comorbid allergy and those without allergy. The results showed no significant differences in serum tTg-IgA concentration or Marsh grade between the two groups. However, there was a significant difference in BMI between the two groups: CeD patients with comorbid allergy had a significantly lower BMI than those without allergy, and diarrhea was more prominent in CeD patients with comorbid allergy (*P* < 0.05). A restrictive diet may be one of the factors contributing to the reduced BMI in CeD patients with comorbid allergy. For patients with CeD, GFD is the only effective treatment, while allergen avoidance is critical for patients with allergies. This dual dietary restriction significantly reduces the variety of foods available to patients, leading to a monotonous dietary structure and consequently causing adverse symptoms such as malabsorption in CeD patients with comorbid allergies. Diarrhea is one of the main clinical manifestations of CeD; GFD treatment typically alleviates diarrhea in CeD patients. However, studies have shown that some patients still experience persistent gastrointestinal symptoms (including diarrhea) after initiating GFD, and comorbid allergies in CeD may be one of the causes of such persistent diarrhea. A retrospective study in Poland found that CeD-related symptoms could persist for up to 2.6 years in CeD patients despite GFD adherence, and approximately 13% of these patients with persistent symptoms had concurrent food and inhalant allergies (52). In a study by Syrigou et al., a CeD patient continued to present with gastrointestinal symptoms and villous atrophy even after 6–12 months of GFD adherence. Subsequent completion of skin prick tests confirmed that the patient had a comorbid soy allergy; after strict adherence to both GFD and a soy-free diet, no symptom recurrence was observed during a 2-year follow-up (53). These studies indicate that comorbid allergies may be one of the factors contributing to persistent gastrointestinal symptoms in CeD patients after GFD initiation. For CeD patients with good GFD adherence but refractory symptoms, comorbid allergies may lead to sustained symptoms, difficulty in restoring nutritional status, and even misdiagnosis as refractory celiac disease (RCD). Therefore, in clinical practice, allergen testing should be incorporated into the routine assessment process for CeD patients with poor response to GFD, to identify potential causes of persistent symptoms at an early stage and reduce the burden on patients.

This study has several limitations. First, the relatively small sample size and the fact that all participants were from a single region (Xinjiang, China) limit the generalizability of the findings. Secondly, in evaluating IgE-mediated allergic conditions, our diagnosis primarily depends on the positivity of specific IgE antibody levels and participants' self-reported allergy history. It should be noted that a positive s-IgE result indicates immunological sensitization but does not necessarily confirm clinical allergy. The absence of objective functional confirmation tests, such as SPT or oral OFC, means that we cannot definitively distinguish between asymptomatic sensitization and true clinical allergy, which may lead to an overestimation of allergy prevalence. In addition, the allergen spectrum is limited by the preset panels of the test kits used. As a result, we did not test for grass pollen-specific IgE, which may restrict our ability to determine whether sensitivity to wheat flour is primary or due to cross-reactivity with pollen. Furthermore, we did not employ component-resolved diagnostics (CRD) to identify reactions to specific allergenic protein components, which could provide additional context for the s-IgE results. Together, these factors may influence the observed sensitization profile and its interpretation. Finally, the accuracy of self-reported data (e.g., allergic rhinitis) may be affected by overlapping symptoms with other respiratory conditions. Therefore, future research with larger sample sizes, multicenter designs, and prospective cohorts is needed to validate the findings of this study.

## 5 Conclusion

To synthesize the above findings, this study indicates that patients with CeD have a significantly higher prevalence of IgE-mediated allergies compared to healthy individuals. Specifically, the most prevalent food allergens in this CeD group are wheat flour, beef, and milk, and the dominant inhalant allergen is dog epithelium. Additionally, comorbid allergies in CeD patients may further aggravate intestinal nutrient absorption disorders and intensify gastrointestinal symptoms, including diarrhea. Consequently, it is suggested that allergen screening followed by specialist allergological evaluation be implemented for CeD patients presenting with severe nutrient malabsorption or intractable diarrhea, to formulate personalized dietary management plans and comprehensive treatment strategies (which extend beyond gluten restriction) for CeD patients with comorbid allergies.

## Data Availability

The original contributions presented in the study are included in the article/Supplementary material, further inquiries can be directed to the corresponding author.
